# Leaf lifespan is positively correlated with periods of leaf production and reproduction in 49 herb and shrub species

**DOI:** 10.1002/ece3.2147

**Published:** 2016-05-09

**Authors:** Fang Lan Li, Xin Liu, Wei kai Bao

**Affiliations:** ^1^ Key Laboratory of Mountain Ecological Restoration and Bioresource UtilizationChengdu Institute of BiologyChinese Academy of SciencesChengduChina

**Keywords:** Functional traits, interspecific relationship, leafing pattern, phenology, plant reproduction, trade‐offs, whole‐plant strategy

## Abstract

Leaf life span and plant phenology are central elements in strategies for plant carbon gain and nutrient conservation. Although few studies have found that leaf life span correlate with the patterns of leaf dynamics and reproductive output, but there have not been sufficient conclusive tests for relationships between leaf life span and plant phenological traits, the forms and strengths of such relationships are poorly understood. This study was conducted with 49 herb and shrub species collected from the eastern portion of the Tibetan Plateau and grown together in a common garden setting. We investigated leaf life span, the periods of leaf production and death, the time lag between leaf production and death, and the period of plant reproduction (i.e., flowering and fruiting). Interspecific relationships of leaf life span with leaf dynamics and reproduction period were determined. Leaf production period was far longer than leaf death period and largely reflected the interspecific variation of leaf life span. Moreover, leaf life span was positively correlated with the length of reproduction (i.e., flowering and fruiting) period. These relationships were generally consistent across different subgroups of species (herbs vs. shrubs) and indicate potentially widely applicable relationships between LLS and aboveground phenology. We concluded that leaf life span is associated not simply with the dynamics of the leaf itself but with reproduction period. The results demonstrate a plant trade‐off in resource allocation between production and reproduction and a coordinated arrangement of leaves, flowers, and fruits in their time investment. Our results provide insight into the relationship between leaf life span and plant phenology.

## Introduction

Leaf life span (LLS) is a key trait involved in fundamental plant trade‐offs between rapid carbon gain and efficient nutrient conservation (Ackerly 1999; Wright et al. 2004; Kikuzawa et al. 2013). LLS should be determined by the time required for a leaf to pay back its construction cost (Chabot and Hicks 1982; Kikuzawa 1991; *Sack* et al. 2013) and then provide a net carbon gain for root, stem, and reproductive organs (Reich et al. 1992; van Ommen Kloeke et al. 2012; Kikuzawa et al. 2013; Edwards et al. 2014). Therefore, plant trade‐offs in resource allocation between leaf growth and reproduction would determine their LLS. Some scientists have found that interspecific variations in LLS correlate with patterns of leaf dynamics (Navas et al. 2003), stem growth, and reproductive output (Rossatto 2013; Edwards et al. 2014; Poorter et al. 2014); however, the forms and strengths of such relationships, as well as their variability among species, are unknown, as the analysis of these relationships is really scarce and restricted to few groups, for example, the Mediterranean vegetation (Navas et al. 2003) and *Viburnum* species (Edwards et al. 2014).

Leaf dynamics is just timing of production and death of leaves and has significant effects on canopy structure and mean LLS of plants (Southwood et al. 1986; Sharma et al. 2012; Rossatto 2013). Dungan et al. (2003) have found the within‐shoot effects of leaf production order on LLS. Presently, we are aware of only one theoretical framework that presents leaf dynamic and their relationships with LLS (Navas et al. 2003). This framework remains unrested. Scientists usually estimate mean LLS based on the changes in the cumulative number of both produced and dead leaves on a particular shoot (Reich et al. 1992; Kikuzawa and Ackerly 1999; Dungan et al. 2003). These demographic data and their relationships to LLS have not been explicitly assessed because of various methodologies (e.g., census interval) applied in each experiment. Monitoring the production and death of leaves on a particular shoot is a precise approach for estimating LLS, but not practical in many studies (Dungan et al. 2003). The question of how leaf dynamics relates to LLS is under debate. Navas et al. (2003) suggested that LLS should be directly related to the period of leaf production, the period of leaf death, and the time lag between the two periods. Results from Mediterranean deciduous species (Navas et al. 2003) and broad‐leaved tree species (Southwood et al. 1986) have indicated that LLS is strongly related to the period of leaf death and the time lag between leaf production and death. Conversely, other studies found that leaf production period potentially interprets variations in LLS (Diemer 1998; Rossatto 2013); hence, the LLS and leaf dynamics relationship is not simple. The relative timing of leaf production and death can affect estimates of mean LLS (Kikuzawa and Ackerly 1999; Navas et al. 2003). Here, we hypothesize that the length of relative period of leaf production and leaf death would determine the LLS–leaf dynamics relationship at the canopy level. Particularly, if the period of leaf production (LP) is longer than the period of leaf death (LD), LP affects the interspecific variations of LLS; alternatively, LD would be a primary influence on LLS if LP is shorter than LD.

In deciduous plants, LLS must be determined by plant phenological traits, which in turn depend on relative proportion of vegetative and reproductive growth. The cost–benefit theory predicts that an optimum LLS depends on both the construction of the leaf itself and the maintenance costs of both structural and reproductive organs (Chabot and Hicks 1982; Kitajima et al. 2013; Sack et al. 2013). Therefore, LLS should be related not simply to the leaf dynamics but to reproduction phenology. The reproduction timing could determine the strength of sinking effect on nutrients stored in the leaves. Theoretically, species with a longer reproduction period would have a larger construction and maintenance cost of reproduction organ than those with shorter carbon investments. That is, species with a long reproduction period still represents a longer carbon investment and would seem to require a longer LLS to achieve a whole‐plant positive carbon balance (Kitajima et al. 2013). Consequently, we hypothesize that LLS is positively related to the period of reproduction. Edwards et al. (2014) have found that LLS was related to peak flowering time in *Viburnum* species. However, there have not been any previous conclusive tests for the relationships between LLS and reproduction period among species. The available data identifying these relationships are relatively scarce and restricted to few groups, for example, *Viburnum*.

Previous studies relating LLS to plant phenology have often compared species measured across sites or study systems (Reich et al. 1992; Diemer et al. 1992; Diemer 1998; Navas et al. 2003; Campanella and Bertiller 2009), leading to confounding of the environmental effects driving the observed relationships (Poorter et al. 2014). To better address these questions, a common garden experiment is needed to understand relationships intrinsic to the plant when individuals are all grown under similar conditions (Reich 2014). This study has an advantage in that all the species were sampled from a common garden with similar nutrient levels, rather than from various habitats as in most previous studies. This particular sampling design may produce some inspiring results and provide insightful viewpoints on the LLS and plant phenology relationship. In this study, 49 deciduous temperate species including two annual herbs, 11 perennial herbs, four perennial subshrubs, and 32 perennial shrubs were monitored in replicated, monoculture plots in a three‐year‐old garden experiment. We recorded definitive LLS, leaf dynamics, and period of plant reproduction and answered two specific questions. (1) How are interspecific variations in LLS related to the periods of leaf production and leaf death of the entire canopy? (2) How do periods of plant reproduction affect LLS? Additionally, we investigated how these patterns varied among different subgroups of deciduous species: annual and perennial herbs, and perennial subshrubs and shrubs.

## Materials and Methods

### Species studied and common garden experiment

This study included 49 deciduous species: two annual herbs, 11 perennial herbs, four perennial subshrubs, and 32 perennial shrubs from diverse families (Table 1). We collected seeds of each species from the natural habitats in the arid Minjiang River valley and its neighboring shrubland in the eastern portion of the Tibetan Plateau (31°42′N, 103°53′E, with altitudes within the range of 1600–1920 m) in autumn 2008. This area has the various microtopography and climatic conditions in natural habitats because of the typical mountainous ecosystem. The species studied grow on soils with various levels of nutrient availability. In the 0‐ to 20‐cm soil layer total nitrogen ranged between 1.95 up to 4.10 g kg^−1^, available phosphorus ranged between 0.81 up to 1.98 mg kg^−1^, available potassium ranged between 0.82 up to 3.15 mg kg^−1^ (Bao et al. 2012).

**Table 1:** The 49 species studied, their life form (following http://foc.eflora.cn/), leaf life span (LLS), period of leaf production (LP), the time lag between the end of leaf production and the start of leaf death (L), period of leaf death (LD), period of plant reproduction (R) and period of plant growth (G). Vine species are indicated by asterisks*. Values are mean ± SE for 5 individuals. *N* = 43 for R, *N* = 49 for other variables

Species	Abbr.	Family	Life form	LLS (days)	LP (days)	L (days)	LD (days)	R (days)	G (days)
*Agrimonia pilosa*	*Apa*	Rosaceae	Perennial herb	124 ± 1.7	145 ± 0.0	−61 ± 0.0	50 ± 2.8	82 ± 0.0	233 ± 1.7
*Ajania potaninii*	*Ap*	Asteraceae	Subshrub	92 ± 1.4	162 ± 1.4	−40 ± 1.4	66 ± 5.2	113 ± 3.4	250 ± 1.4
*Anaphalis gracilis*	*Ags*	Asteraceae	Subshrub	107 ± 1.4	159 ± 1.7	−58 ± 1.5	50 ± 2.8	128 ± 0.0	242 ± 1.7
*Artemisia gmelinii*	*Ag*	Asteraceae	Subshrub	101 ± 3.4	131 ± 1.7	−42 ± 1.4	51 ± 2.8	139 ± 2.8	206 ± 0.0
*Artemisia roxburghiana*	*Ar*	Asteraceae	Perennial herb	107 ± 5.2	147 ± 1.7	−45 ± 1.4	33 ± 2.8	166 ± 3.5	217 ± 1.7
*Aster albescens*	*Aa*	Asteraceae	Subshrub	160 ± 1.7	174 ± 1.7	−37 ± 2.8	36 ± 2.8	102 ± 3.1	229 ± 1.4
*Bauhinia brachycarpa*var*. microphylla*	*Bb*	Fabaceae	Shrub	232 ± 1.7	265 ± 0.0	−75 ± 1.7	43 ± 2.8	_	323 ± 1.7
*Berberis diaphana*	*Bd*	Berberidaceae	Shrub	153 ± 1.4	200 ± 2.8	−56 ± 0.0	50 ± 1.7	168 ± 8.1	277 ± 2.8
*Berberis wilsonae*	*Bw*	Berberidaceae	Shrub	132 ± 1.4	205 ± 1.4	−49 ± 0.0	34 ± 1.7	167 ± 6.3	257 ± 3.4
*Buddleja davidii*	*Bdi*	Loganiaceae	Shrub	162 ± 2.8	165 ± 1.4	−90 ± 1.4	36 ± 1.4	168 ± 4.7	252 ± 1.4
*Campylotropis polyantha*	*Cpa*	Fabaceae	Shrub	178 ± 1.4	188 ± 1.7	−63 ± 1.7	50 ± 1.4	128 ± 6.0	253 ± 1.4
*Campylotropis wilsonii*	*Cw*	Fabaceae	Shrub	152 ± 0.0	158 ± 1.7	−44 ± 1.4	34 ± 2.8	208 ± 6.1	211 ± 2.8
*Caryopteris tangutica*	*Ct*	Verbenaceae	Shrub	107 ± 1.4	144 ± 2.8	−60 ± 1.4	30 ± 1.7	119 ± 4.7	202 ± 2.8
*Ceratostigma illmottianum*	*Cm*	Plumbaginaceae	Shrub	138 ± 0.0	159 ± 0.0	−75 ± 0.0	30 ± 0.0	144 ± 5.4	240 ± 1.7
*Clematis potaninii*	*Cp*	Ranunculaceae	Perennial herb*	105 ± 1.7	131 ± 2.8	−52 ± 1.4	51 ± 1.7	100 ± 6.1	225 ± 1.4
*Corylus heterophylla*var.*sutchuenensis*	*Chv*	Betulaceae	Shrub	171 ± 1.4	177 ± 3.4	−50 ± 1.4	40 ± 5.2	_	261 ± 2.7
*Cotinus szechuanensis*	*Cs*	Ranunculaceae	Shrub	138 ± 1.4	146 ± 1.4	−45 ± 0.0	65 ± 1.7	54 ± 2.9	198 ± 5.1
*Cotoneaster hebephyllus*	*Chs*	Rosaceae	Shrub	220 ± 1.4	220 ± 2.8	−105 ± 1.4	94 ± 3.4	165 ± 5.4	360 ± 2.8
*Cotoneaster horizontalis*	*Ch*	Rosaceae	Shrub	156 ± 1.4	203 ± 1.7	−47 ± 1.7	34 ± 2.8	141 ± 6.1	276 ± 0.0
*Cryptotaenia japonica*	*Cj*	Apiaceae	Perennial herb	92 ± 2.8	101 ± 0.0	−33 ± 1.7	66 ± 2.8	72 ± 5.3	175 ± 1.7
*Echinochloacrusgali*var*. mitis*	*Ecv*	Poaceae	Perennial herb	120 ± 1.4	146 ± 1.4	−40 ± 1.4	50 ± 0.0	59 ± 5.1	220 ± 1.4
*Elaeagnus stellipila*	*Es*	Elaeagnaceae	Shrub	151 ± 1.4	155 ± 5.1	−53 ± 2.8	80 ± 2.8	196 ± 3.8	241 ± 1.4
*Geranium nepalense*	*Gn*	Geraniaceae	Perennial herb	80 ± 1.4	95 ± 1.7	−40 ± 1.4	39 ± 2.8	_	167 ± 1.7
*Hylodesmum podocarpum*	*Hpo*	Fabaceae	Shrub	167 ± 1.7	183 ± 1.7	−54 ± 1.4	64 ± 1.4	87 ± 4.1	227 ± 1.4
*Hyperlcurn perforatum*	*Hpe*	Clusiaceae	Perennial herb	102 ± 1.4	113 ± 1.4	−35 ± 2.8	50 ± 1.4	123 ± 5.9	257 ± 1.4
*Indigofera amblyantha*	*Ia*	Fabaceae	Shrub	135 ± 1.4	140 ± 1.7	−50 ± 1.7	30 ± 3.4	208 ± 6.0	224 ± 0.0
*Indigofera silvestrii*	*Isi*	Fabaceae	Shrub	155 ± 0.0	168 ± 1.7	−73 ± 1.4	30 ± 2.8	188 ± 7.5	245 ± 3.4
*Indigofera szechuensis*	*Is*	Fabaceae	Shrub	149 ± 1.4	191 ± 2.8	−39 ± 1.7	80 ± 1.7	192 ± 6.8	239 ± 2.8
*Kerria japonicus*	*Kj*	Rosaceae	Shrub	202 ± 1.5	221 ± 1.4	−73 ± 2.8	35 ± 3.4	196 ± 0.0	317 ± 7.1
*Lespedeza daurica*	*Ld*	Fabaceae	Shrub	137 ± 1.5	139 ± 1.4	−45 ± 1.4	35 ± 1.7	124 ± 3.6	202 ± 1.4
*Lespedeza formosa*	*Lf*	Fabaceae	Shrub	126 ± 2.8	162 ± 1.7	−30 ± 1.4	51 ± 0.0	157 ± 7.1	189 ± 1.7
*Lonicera japonica*	*Lj*	Caprifoliaceae	Shrub*	125 ± 1.4	161 ± 1.4	−76 ± 1.4	48 ± 0.0	95 ± 2.8	200 ± 1.7
*Lycium chinense*	*Lc*	Solanaceae	Shrub	123 ± 1.4	152 ± 0.0	−51 ± 1.4	86 ± 1.7	156 ± 6.5	203 ± 1.5
*Ranunculus japonicus*	*Rj*	Ranunculaceae	Perennial herb	68 ± 0.0	102 ± 1.7	−37 ± 1.7	55 ± 1.7	61 ± 3.4	150 ± 2.8
*Rhynchosia minima*	*Rma*	Fabaceae	Annual herb	53 ± 1.4	86 ± 2.8	20 ± 0.0	54 ± 1.4	111 ± 0.0	157 ± 1.4
*Rosa filipes*	*Rf*	Rosaceae	Shrub	165 ± 1.4	185 ± 0.0	−44 ± 0.0	88 ± 1.4	170 ± 6.2	255 ± 1.7
*Rosa hugonis*	*Rh*	Rosaceae	Shrub	180 ± 1.7	200 ± 2.8	−73 ± 1.7	80 ± 1.7	83 ± 2.8	269 ± 0.0
*Rosa multibracteata*	*Rm*	Rosaceae	Shrub	170 ± 1.7	198 ± 1.4	−60 ± 2.8	64 ± 3.4	152 ± 4.9	245 ± 2.8
*Rosa sericea*	*Rsa*	Rosaceae	Shrub	163 ± 1.4	180 ± 1.4	−36 ± 2.8	61 ± 1.7	170 ± 2.8	223 ± 1.7
*Rosa soulieeana*	*Rs*	Rosaceae	Shrub	127 ± 2.8	142 ± 2.8	−60 ± 1.7	50 ± 3.4	159 ± 2.8	227 ± 1.7
*Rubus pungens*var*. ternatus*	*Rpv*	Rosaceae	Shrub*	174 ± 1.4	180 ± 1.4	−35 ± 1.7	79 ± 1.4	80 ± 2.8	278 ± 1.4
*Rubus setchuenensis*	*Rss*	Rosaceae	Shrub*	124 ± 1.4	154 ± 1.4	35 ± 2.8	80 ± 2.8	62 ± 0.0	231 ± 1.4
*Setaria viridis*	*Sv*	Poaceae	Annual herb	72 ± 1.7	101 ± 1.7	−20 ± 1.4	41 ± 1.7	47 ± 2.1	136 ± 5.2
*Sophora davidii*	*Sdi*	Fabaceae	Shrub	206 ± 2.8	269 ± 0.0	−93 ± 1.4	62 ± 0.0	_	296 ± 3.4
*Sorbaria arborea*	*Sa*	Rosaceae	Shrub	168 ± 1.4	182 ± 2.8	−43 ± 1.4	53 ± 1.7	_	258 ± 5.1
*Spiraea dahurica*	*Sd*	Rosaceae	Shrub	180 ± 1.4	200 ± 1.4	−62 ± 0.0	46 ± 2.8	124 ± 2.8	273 ± 3.4
*Vicia cracca*	*Vc*	Fabaceae	Perennial herb*	83 ± 1.4	124 ± 1.7	−15 ± 2.8	36 ± 1.77	137 ± 2.8	158 ± 2.8
*Vicia taipaica*	*Vt*	Fabaceae	Perennial herb*	63 ± 1.4	131 ± 2.8	−42 ± 1.7	54 ± 7.4	116 ± 5.1	213 ± 5.2
*Vicia unijuga*	*Vu*	Fabaceae	Perennial herb	136 ± 2.8	140 ± 1.7	−66 ± 1.7	40 ± 1.4	116 ± 2.8	210 ± 1.7

After natural air‐drying for 4–8 days, seeds of Rosaceae species were scarified with 98% H_2_SO_4_ for 4 h and then were stratified at 5°C for 8 weeks to break seed dormancy. Other seeds were stored at room temperature (10–25°C) until sowing. Before sowing, all seeds were disinfected by immersion in 2.5% NaClO for 1 h. Perennial species were grown in a garden from spring 2009 until the end of the study in 2011. Annual species were established in the experimental plots on 10 March 2011.

The study was conducted in a common garden at the Maoxian Mountain Ecosystem Research Station (MMERS) operated by the Chinese Academy of Sciences, located in Maoxian County, Sichuan, China (31°41′N,103°53′E, altitude 1830 m). The slope across the common garden was <10°. Climate is temperate semi‐arid; monthly climatic data, including mean rainfall, air temperature, evapotranspiration, air humidity, and hours of sunshine during the study period (2009–2011) were provided by the MMERS Meteorological Observatory. This site experiences average annual precipitation of 738.8 mm and approximately 70% of this occurs in the rainy season of May–October. Annual mean temperature is 10.4°C, with average temperatures of 18.7°C and −1.4°C in July and January, respectively. The common garden soil was characterized as Udic luvisol with uniform physical and chemical properties over the entire field. The 0‐ to 20‐cm soil layer was characterized by organic matter of 17.22 ± 2.02 g kg^−1^, total nitrogen of 1.64 ± 0.12 g kg^−1^, available phosphorus of 9.19 ± 1.45 mg kg^−1^, available potassium of 8.25 ± 9.01 mg kg^−1^, pH 5.45 ± 0.11, and bulk density 0.97 ± 0.10 g cm^−3^. Soil water content was about 18% during the rainy season and about 7% in the dry season.

The experiment had a randomized block design with each of the 49 species repeated in five blocks. We established 165 plots (2 m × 2 m) for shrub species and 80 plots (1 m × 1.5 m) for subshrub and herbaceous species on 1 April 2009. For each species, 20 seeds of similar size were sown in each of five plots. Shortly after emergence, seedlings of all species were thinned and 10 average‐sized plants per plot were kept. Distance between the plants was around 60–80 cm in each plot. All plots were initially well watered and shaded to facilitate seedling establishment. The garden was not fertilized during the experiment, but was weeded manually twice each year to reduce competition by other plants.

All observations were performed in 2011. Prior to spring emergence on 10 March 2011, we selected one average‐sized individual in each of five plots and marked three well‐lit branches per individual with plastic labels. Each of these branches was observed repeatedly to measure patterns of LLS, leaf dynamics, and reproduction phenology.

### Measurement of LLS

Three buds of leaves or leaf cohorts were selected on the middle part of each tagged branch for a census of emergence and fall and for the determination of LLS. For most observed species in this study, LLS of each tagged individual leaf was calculated directly based on the date of leaf emergence and fall. For some species such as *Ajania potaninii, Berberis diaphana*,* B. wilsonae, Lycium chinense, Sophora davidii*, with small and fasciated individual leaves that is difficult to tag and record for each leaves, hence the leaf cohort was recorded because of practical reason. We have defined a leaf cohort as leaves growing at the same internode and emerging at the same time, which accords with the concept of the leaf cohort, that is, any leaves emerging together at some time form an even‐aged cohort which concepts as leaf cohort (Kikuzawa and Lechowicz 2011). Observations were conducted biweekly until all leaves forming observed cohorts for a given species had senesced. The date of emergence and the date of fall for each leaf or cohort were noted. From the two dates, the absolute LLS was calculated as duration from leaf emergence to leaf fall for each marked leaf or cohort.

### Measurements of leaf dynamics and plant phenology

At each observation date of leaf dynamics, we marked all present leaves using spray lacquer on the petioles and noted the total number of living and dying leaves remaining on the stem since the previous census. We also performed a regular census of all marked branches and noted plant phenophases using the methods and definitions outlined in Haggerty and Mazer (2008). The following parameters were recorded: (1) onset of greening (first leaf was fully expanded); (2) first flower; (3) fruit fully ripe (fruit maturity and seed dispersal); and (4) leaf senescence (color change and abscission). Finally, period of leaf production (LP, the time from the appearance of first leaf to the last), period of leaf death (LD, the time from the first fallen leaf to the last), and the time lag between leaf production and death (L, the time from the appearance of last leaf to the first fallen leaf), period of plant reproduction (R, the time from the first flower to fruit maturity and seed dispersal), and period of plant growth (G, the time from onset of greening to leaf senescence) were evaluated. Five species did not produce flowers during the study period (Table 1); thus, reproduction period was not taken into account in our investigation.

### Statistical analyses

We calculated means and standard errors of all traits for each species using individual plots as the unit of replication (*n* = 5). To detect the effects of life form on LLS, leaf dynamics, and period of plant reproduction, differences among the three plant groups, annual and perennial herbs, subshrubs, and shrubs, were analyzed using one‐way ANOVA. The least significant differences (LSD) between the means were estimated at 95% confidence level.

Relationships between LLS and other variables across species of each plant form and across all species were determined with linear regression analyses. As no significant effects of plant forms (above three groups) and ontogenetic (flowered or not) were found on the trait–trait relationships, all regression and correlation analyses were performed on the whole dataset, and all species were separated into two groups as either herb (include subshrub) or shrub species. Differences in the intercept and slope of the linear regression equations for each pair of variables between two groups were then tested with analysis of covariance (ANCOVA). Pearson's correlation analyses were performed on the whole dataset to examine the trait‐by‐trait interrelationships. All of these statistics and significance tests were conducted using SPSS (version 16.0, SPSS Inc., Chicago, IL).

Phylogenetic independent contrasts (PICs) were performed in the R software (using the picante package). The phylogenetic tree for 49 species was constructed using the Phylomatic utility, based on the APG III and Flora of China. PICs were also performed to analyze the evolutionary relationships between a set of traits. A principal component analysis (PCA) was performed to determine the correlations among the observed traits for the 49 species and to determine the species' trait syndromes using the R software using the picante package and vegan package (http://CRAN.R-project.org/package=vegan).

## Results

### Patterns of leaf dynamics and LLS

**Figure 1:**
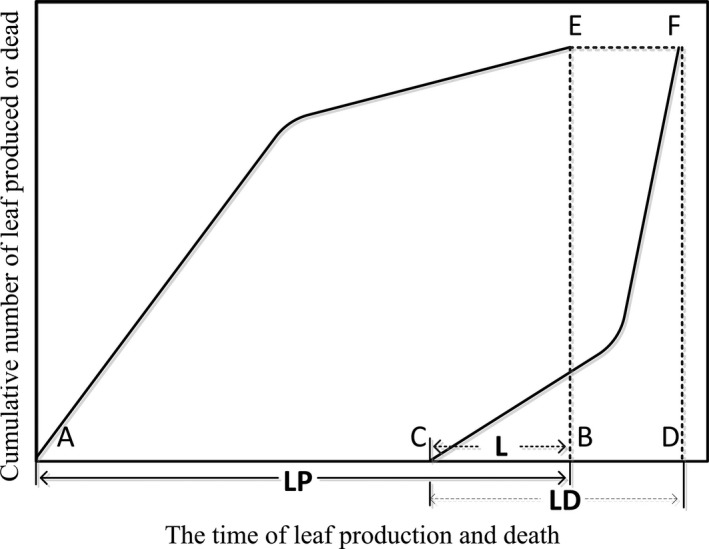
Conceptual model describing leaf dynamics through the growing season. Each letter represents a key phenological date as follows: A, first leaf production; B, last leaf production; C, first leaf death; D, last leaf death. E and F, theoretical total leaf number. Dotted lines represent the end of the period of leaf production [BE] and end of period of leaf death [DF]. Solid lines link the cumulative number of leaves produced [AE] or lost [CF] through time. LP is the period of leaf production (solid arrow), LD is the period of leaf death (dashed arrow) and L is the time lag between the end of leaf production and the start of leaf death (dashed and dotted arrow).

We observed an extensive overlap between LP and LD (Fig. 1), with LP much longer than LD (Table 1). LLS was much longer for the shrub group than for the annual herbs (*P *<* *0.001, *F *=* *17.29), whereas perennial herbs and subshrub species were intermediate and did not differ significantly from the other plant forms (*P *>* *0.05, one‐way ANOVA).

### Relationships of LLS to leaf dynamics and reproduction period

**Figure 2:**
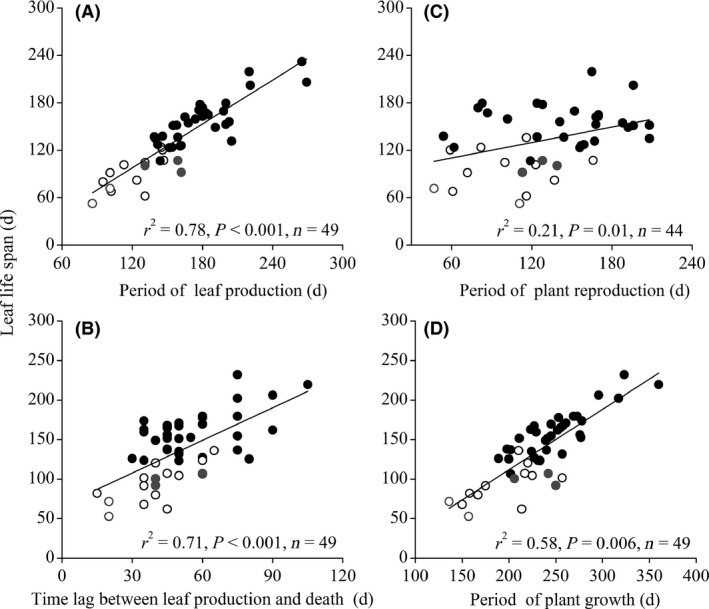
Relationships between leaf life span (LLS) and (A) period of leaf production (LP), (B) time lag between the end of leaf production and the start of leaf death (L), (C) period of plant reproduction (R) and (D) period of plant growth (G) for the considered species: gray open circle, annual herb; black open circle, perennial herb; gray solid circle, subshrub; black solid circle, shrub. The regression line is fitted to all species.

Leaf life span was positively related to LP (*P *<* *0.01, *r* ^2 ^= 0.78, *F *=* *165.49; Fig. 2A) and L (*P *<* *0.01, *r* ^2^ = 0.71, *F *=* *132.93; Fig. 2B), but was not related to LD (*P *=* *0.58, *r* ^2^ = 0.07, *F *=* *0.32, data not shown). There was a significant positive interrelationship between L and LP (*r = *0.61, *P *<* *0.01), whereas L and LD were unrelated (*r = *−0.0.6, *P *>* *0.05, data not shown). LLS was positively related to R (*P *<* *0.01, *r* ^2^ = 0.28, *F *=* *17.14; Fig. 2C) and G (*P *=* *0.01, *r* ^2^ = 0.15, *F *=* *6.87; Fig. 2D). Slopes of the regression lines of these observed relationships did not differ between herbaceous and woody species (*P *=* *0.71–0.86, ANCOVA, Table 2). When PICs were included in the Pearson's correlations, the patterns of interrelationships among traits were conserved (Table 3), indicating that species evolutionary history did not influence the detected relationships (*K < *1, *P <* 0.05).

**Table 2:** Linear regression analysis between leaf life span (LLS) and leaf dynamics traits, plant phenological traits of herb (include subshrubs) and shrub groups. Slope, coefficient of determination (*r* ^2^) and significance level (***P <* *0.001;**P <* *0.01; *P <* *0.05; *N* = 43 for R, *N* = 49 for other variables) of the linear regression equation are shown for herb and shrub species separately. *P*‐value in the last column is significance level of ANCOVA for the regression slopes of the herb and shrub species. See Table 1 for trait abbreviations

Trait	Herb		Shrub		*F*‐value	*P*‐value
	Slope	*r* ^2^	Slope	*r* ^2^		
LP	0.96	0.53**	0.88	0.70***	0.14	0.71
L	0.32	0.20*	0.29	0.20**	0.02	0.86
LD	−0.13	0.02	−0.07	0.02	–	–
R	0.18	0.00	0.06	0.03	–	–
G	0.71	0.25*	0.74	0.27**	0.10	0.79

**Table 3:** Correlation matrix among leaf life span (LLS) and plant phenological traits with tests of correlation coefficient and significance level (***P <* *0.001;**P <* *0.01; *P <* *0.05; *N* = 43 for R, *N* = 49 for other variables), provided by the Pearson Correlation Analyses. Above diagonal is Pearson Correlation Analyses and below diagonal is based on phylogenetically independent contrasts. See Table 1 for trait abbreviations

	LLS	LP	L	LD	R	G
LLS		0.86***	0.62**	0.05	0.41**	0.79***
LP	0.73***		0.61***	0.11	0.44**	0.52***
L	0.76***	0.37**		−0.05	0.35*	0.50***
LD	0.28	0.23	−0.06		−0.27	0.10
R	0.45**	0.45**	0.37*	−0.27		0.29
G	0.85***	0.70***	0.80***	0.10	0.29	

“−” indicates negative correlation.

**Figure 3:**
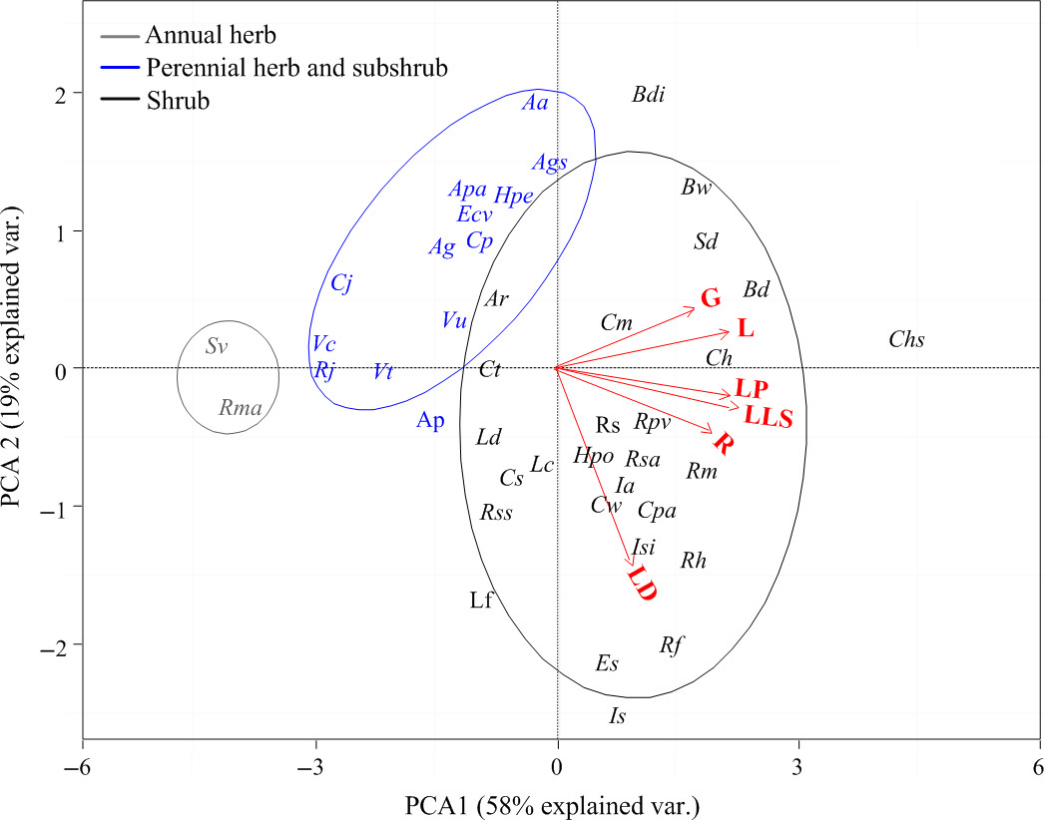
Display of species traits along the first two PCA axes. Trait (roman) and species (italic) abbreviations are given in Table 1.

The first two axes of the PCA explained 77% of the variability (PCA1: 58%, PCA2: 19%, Fig. 3). LP, LLS, R, and G made a strong contribution to axis 1. The first axis generally represents a gradient of species from those with successive leaf appearance, long LLS, R and G to flushing appearance, short LLS, R and short G. Axis 2 was principally related to LD. Thus, the second axis represents a gradient of species from those with short LD (flushing leaf fall) to long LD (successive leaf fall).

## Discussion

In this study, the pattern of leaf dynamics of observed species differs from the previous theoretical framework developed in similar Mediterranean ecosystems (Navas et al. 2003). One prominent difference among the graphical frameworks is a relative timing of leaf production and loss in leaf dynamics. Another significant distinction among the different graphical frameworks is the values of the length of the period from the end of leaf production to the start of leaf loss. In this study, the leaves that produce initially in early spring and they successively until late summer, causing a relatively long LP compared with LD and presented an alternative framework of leaf dynamics (Fig. 1). Therefore, these species can be considered as successive leafing species providing an explanation for a relationship between LLS and LP (see below section). Moreover, in our framework the increase (leaf production) and decrease (leaf death) in the cumulative numbers of new leaves were reflected by curves over time (Fig. 1), which does not support for “line growing” framework of Navas et al. (2003). We found that individual leaves or cohorts produce one by one over a long period and then flush fall all leaves in a short period. The peaks of leaf production and death of many species occurred in early July and late October, respectively. Our result is supported by the findings of Campanella and Bertiller (2009) for shrubs and of Sharma et al. (2012) for mangrove species. Kikuzawa (1995) suggested leaf phenology and lifespan must be the central elements in any strategy of plant carbon gain of plant. This pattern may be associated with the evolutionary adaptation of plants to the seasonally dry and windy climate in the arid valleys of the eastern Tibetan Plateau (Li and Bao 2014). Plants usually adopt this pattern of successive leafing over a long period (i.e., multiple leaf flushes), while they have a single, relatively concise period of leaf senescence in unstable environments (Kikuzawa 1995).

Many species appear to have a time overlap between leaf production and death (i.e., L is a negative number) due to successive leafing (Fig. 1). As new leaves are produced in the upper canopy, the older and shaded leaves are often lost in the lower canopy. Negative L values were found in Mediterranean ecosystems (Navas et al. 2003) and temperate grasslands (Craine et al. 1999) and may be explained by the self‐shading model which predicts a decrease in photosynthetic capacity with leaf age (Kikuzawa 1991; Ackerly and Bazzaz^1995^; Mediavilla and Escudero 2003). Plants with successive leaf emergence utilize full sunlight effectively. Therefore, this pattern of leaf dynamics could partly compensate for decreasing carbon gain resulting from self‐shading and/or leaf death and is considered a strategy for favorable carbon gain of plants (Kikuzawa and Lechowicz 2011). In addition to this self‐shading hypothesis, the redistribution of resources to younger leaves leading to shedding of older leaves has been found in some evergreen woody species (Mediavilla and Escudero 2003; Marty et al. 2010). Leaf shedding pattern may be controlled by both carbon assimilation function and nutrient storage function of leaves (Marty et al. 2010). During leaf production process, nutrients are transferred from old to young leaves to support their growth. In present study, LLS was positively related to LMA, whereas was negatively related to [N], and the scatter spots of the two regression relationships lie within the range of the dataset in global leaf economic spectrum **(**Appendix S1). We independently drew attention to the mechanism underlying ecological strategy using leaf dynamic traits as main factor for interpretation of the relationships of LLS to leaf [P]. Species with higher leaf [P] will have longer LLS (Appendix S2). Sufficient P is associated with cytokinin levels in plants (Güsewell 2004; Han et al. 2005; Ågren 2008), which can promote leaf production and shoot growth, whereas P deficiency can accelerate leaf senescence and decrease LLS (Güsewell 2004).

As predicted, the LLS–leaf dynamics relationships were directly related to the length of the relative period of leaf production and death. LP was far longer and was more positively related to LLS than their respective LD (Fig. 2), which provides evidence supporting our hypothesis that LP is the primary influence on LLS. This finding contrasts with that reported in other ecosystems (Southwood et al. 1986; Navas et al. 2003). For example, plant species from high elevation (4000–4600 m a.s.l.) grasslands in the Andes (Diemer 1998), arctic ecosystems (Prock and Körner 1996), and Mediterranean regions (Navas et al. 2003) have longer LD that are more related to LLS than LP. These differences in relationships between leaf phenology and LLS have been related to changes in branching patterns and shoot growth rate. Furthermore, these various LLS–leaf dynamics relationships are due not only to the ecological strategies of different related species but also to various environmental factors in these different ecosystems (Kikuzawa et al. 2013; Reich 2014). In addition to LP, the L‐LLS relationship would also be significant (Fig. 2B). These results suggest that the deciduous species with a fixed growth season will achieve highest average LLS on canopy by continually producing leaves during growing season, and maximizing the lag time between leaf production and leaf death.

In each of these deciduous plants, particular LLS was correlated with other plant traits. We found a positive relationship between LLS and R (Fig. 2C). It indicates that LLS is linked to the cost or timing of reproductive organs. This finding provides new evidence for the cost–benefit model of LLS that an optimum LLS depends on both the construction and maintenance costs of the leaf itself (Kikuzawa and Ackerly 1999; Sack et al. 2013) and other plant organs (Chabot and Hicks 1982). Species with greater carbon investments in reproduction would have longer LLS those with lesser investments (Kitajima et al. 2013). Edwards et al. (2014) found that LLS was significantly related to flowering time in woody *Viburnum* species. These results demonstrate that LLS and reproduction period can be tightly coupled at the whole‐plant level. A common slope between herbaceous and woody groups in each observed relationship suggests that the LLS–reproduction period relationships varied little between herbaceous and woody plant groups in this common environment. This is consistent with other leaf economic spectrum traits (Osnas et al. 2013; Price et al. 2014). The length of reproduction period alone could not be considered to be the cost of reproduction. Other factors such as number of flowers, amount of nectar, ovary and fruit, and seed sizes, all contribute to the cost of reproduction. Therefore, more works should be done to test the relationships between LLS and these reproduction traits in details.

Phenology trait syndromes using PCA analysis mainly enabled the ranking of species along a gradient from the successive leafing species (perennial shrubs), which display long LLS, R, and G, to flushing species (annual herbs), which display short LLS, R and short G (Fig. 3), revealing a trade‐off between efficient nutrient conservation and high carbon gain. The positive correlations between LLS and the percentage of support tissue and stem density provide a theoretical explanation for why LLS different from among the growth forms. We detected the differences in LLS among the growth forms in a common environment. These LLS variations were related to different levels of the carbon stocks of both supporting tissues, but not to plant size (Appendix S3). Moreover, these differences exhibited among the growth forms indicate that the observation period most likely represents different phases of the total life span of short‐lived annual herbs and long‐lived perennial shrubs. Perennial herbs and subshrub species showed intermediate values of observed traits.

In conclusion, we found support for the first hypothesis that the length of the relative period of leaf production and death was the main factor affecting the LLS–leaf dynamics relationships. Leaf production period was consistently longer than leaf death period and hence had a greater effect on LLS. Furthermore, the interspecific variation in LLS was closely related to the reproduction period of plants, which provides important evidence supporting the second prediction that reproductive phenology can influence LLS at the whole‐plant level. These results underline the importance for researchers to consider LLS–plant phenology relationships to better understand patterns of LLS across species in future studies.

## Conflict of Interest

None declared.

## Supporting information


**Appendix S1.** Relationships between leaf life span (LLS) and (a) leaf mass per area (LMA) and (b) leaf nitrogen concentration [N] for the considered species and within the global LES trait space.
**Appendix S2.** Relationships between leaf life span (LLS) and leaf phosphorus concentration [P] for the considered species: grey open circle, annual herb; black open circle, perennial herb; grey solid circle, subshrub; black solid circle, shrub.
**Appendix S3.** Relationships between leaf life span (LLS) and (a) biomass[BM], (b) coverage height, (c) percentage of supporting tissues and (d) stem density for the considered species: grey open circle, annual herb; black open circle, perennial herb; grey solid circle, subshrub; black solid circle, shrub.
