# Supplementary material for: Leaf lifespan is positively correlated with periods of leaf production and reproduction in 49 herb and shrub species
Source: Ecol Evol. 2016 May 9;6(11):3822–31. doi: 10.1002/ece3.2147 (PMC4933094; doi:10.1002/ece3.2147)
Supplement: Supplementary file 1 — Appendix S1. Relationships between leaf life span (LLS) and (a) leaf mass per area (LMA) and (b) leaf nitrogen concentration [N] for the considered species and within the global LES trait space. Appendix S2. Relationships between leaf life span (LLS) and leaf phosphorus concentration [P] for the considered species: grey open circle, annual herb; black open circle, perennial herb; grey solid circle, subshrub; black solid circle, shrub. Appendix S3. Relationships between leaf life span (LLS) and (a) biomass[BM], (b) coverage height, (c) percentage of supporting tissues and (d) stem density for the considered species: grey open circle, annual herb; black open circle, perennial herb; grey solid circle, subshrub; black solid circle, shrub. [file ECE3-6-3822-s001.docx]

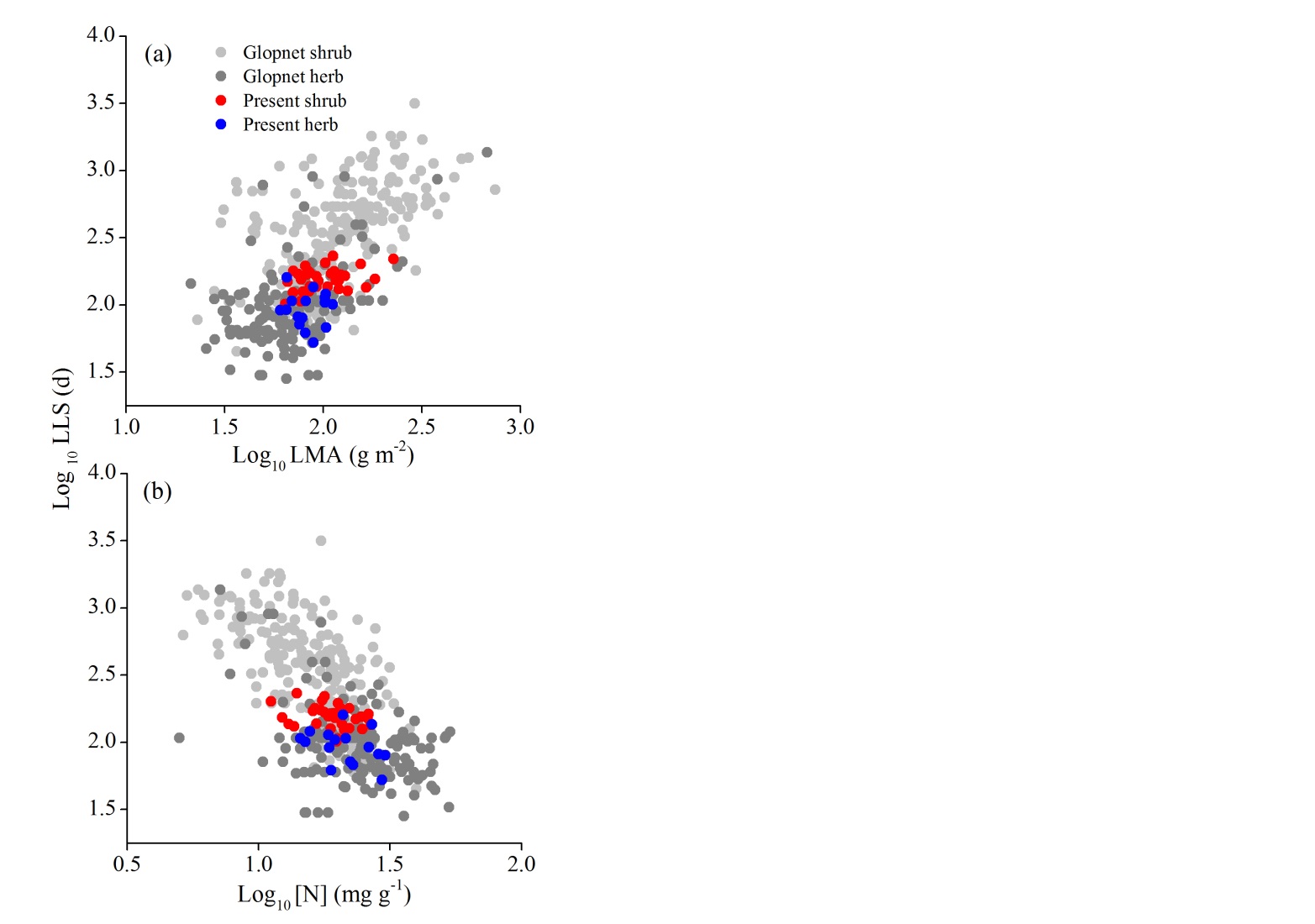


**Appendix S1** Relationships between leaf life span (LLS) and (a) leaf mass per area (LMA) and (b) leaf nitrogen concentration [N] for the considered species and within the global LES trait space. The original ‘glopnet’ data set of shrub and her species used in Wright et al. (2004). LLS, Leaf life span; LMA, leaf mass per area; [N], nitrogen concentration per leaf mass.


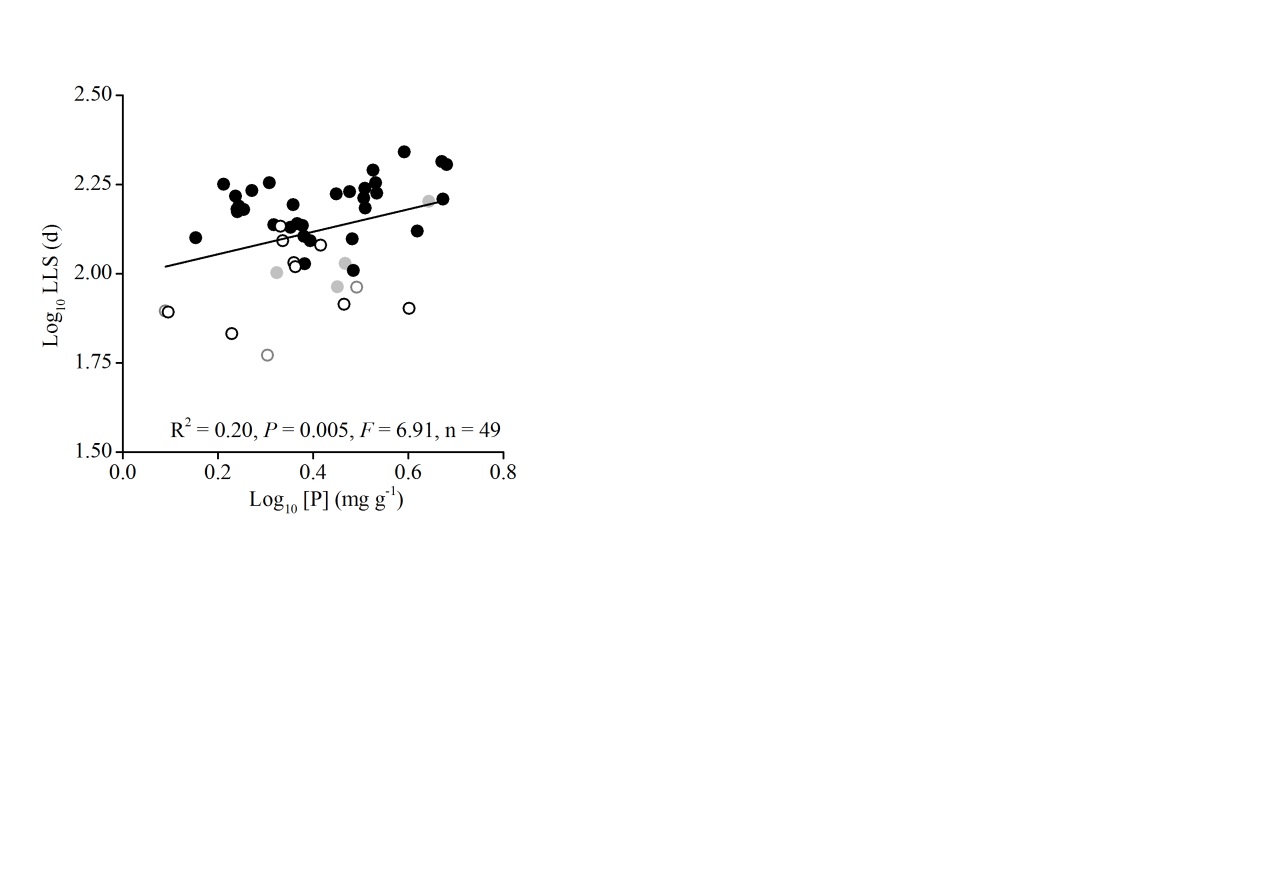


**Appendix S2** Relationships between leaf life span (LLS) and leaf phosphorus concentration [P] for the considered species: grey open circle, annual herb; black open circle, perennial herb; grey solid circle, subshrub; black solid circle, shrub. The regression line is fit to all species. Coefficient of determination (*R*^2^) and *P* and *F* level are shown.


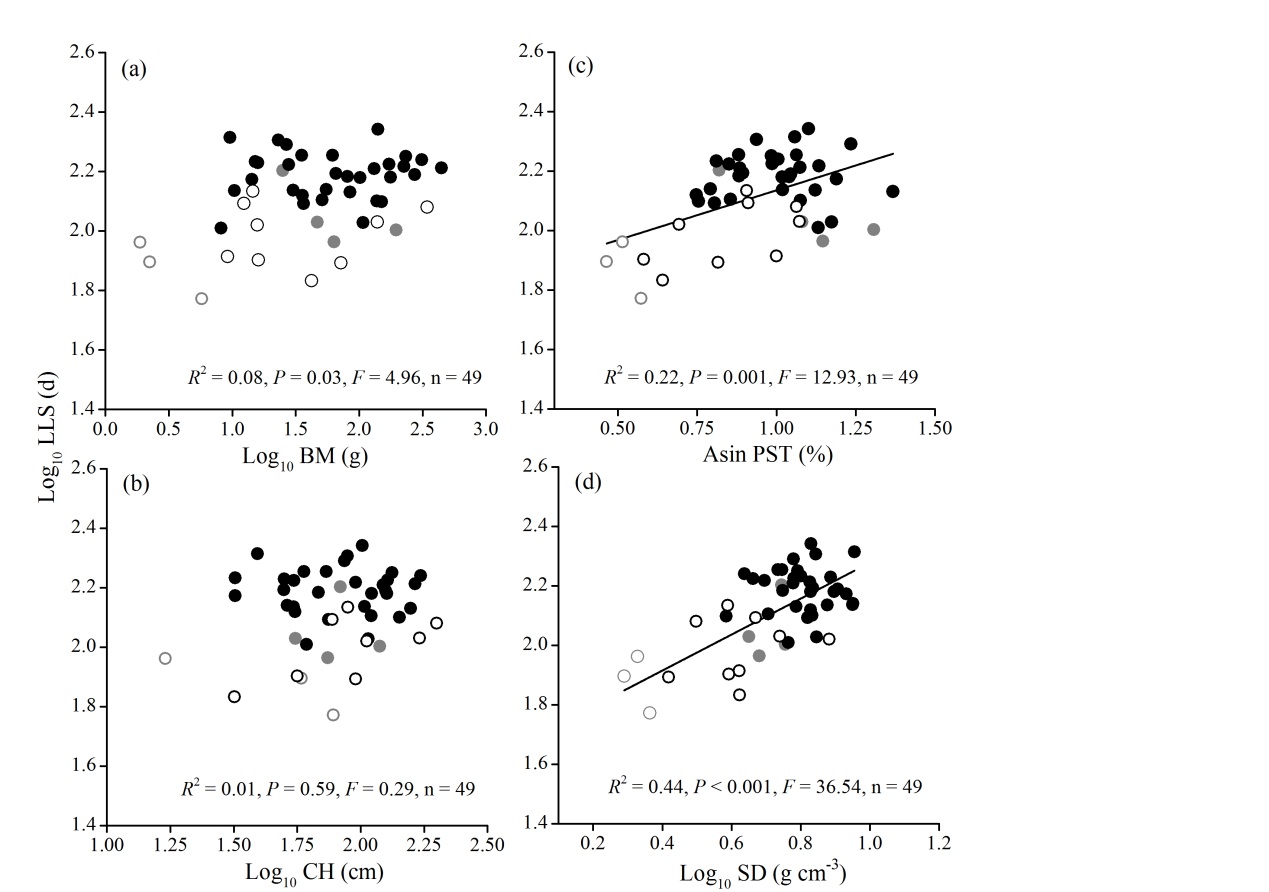


**Appendix S3** Relationships between leaf life span (LLS) and (a) biomass[BM], (b) coverage height, (c) percentage of supporting tissues and (d) stem density for the considered species: grey open circle, annual herb; black open circle, perennial herb; grey solid circle, subshrub; black solid circle, shrub. The regression line is fit to all species. Coefficient of determination (*R*^2^) and *P* and *F* level are shown.
